# Modifications of Biodentine and Their Influence on Endodontic Properties: A Scoping Review

**DOI:** 10.1155/ijod/4886436

**Published:** 2026-06-17

**Authors:** Rumesa Batul, Abdul Habeeb Adil, Niher Tabassum Snigdha, Ankita Mathur, Sushma Bommanavar, Mohmed Isaqali Karobari

**Affiliations:** ^1^ Conservative Dentistry Unit, School of Dental Sciences, University Sains Malaysia, Health Campus, Kota Bharu, 16150, Malaysia, usm.my; ^2^ Centre of Research Impact and Outcome, Chitkara University, Rajpura, 140417, Punjab, India, chitkara.edu.in; ^3^ Centre for Innovation and Inclusive Research, Sharda University, Greater Noida, Uttar Pradesh, India, sharda.ac.in; ^4^ Department of Dental Research, Saveetha Medical College and Hospital, Saveetha Institute of Medical and Technical Sciences, Saveetha University, Chennai, Tamil Nadu, 602105, India, saveetha.com; ^5^ Department of Orthodontics, Pedodontics and Preventive Dentistry, Faculty of Dentistry, Ibn Al-Nafis University, Sanaa, Yemen; ^6^ Department of Dental Research Cell, Dr. D. Y. Patil Dental College and Hospital, Patil Vidyapeeth (Deemed to be University), Pimpri, Pune, 411018, India, dpu.edu.in; ^7^ Centre for Research and Innovation, Academy of Maritime Education and Training (AMET) Deemed to be University, Kanathur, Chennai, 603112, Tamil Nadu, India, ametuniv.ac.in; ^8^ Department of Conservative Dentistry and Endodontics, Saveetha Dental College and Hospital, Saveetha Institute of Medical and Technical Sciences, Saveetha University, Chennai, Tamil Nadu, 602105, India, saveetha.com; ^9^ Department of Conservative Dentistry and Endodontics, Faculty of Dentistry, University of Puthisastra, Phnom Penh, 12211, Cambodia, puthisastra.edu.kh

**Keywords:** additive, bioceramic material, Biodentine, biological property, calcium silicate cement, mechanical property, modification, nanoparticle, physical property, tricalcium silicate

## Abstract

**Background:**

Biodentine, an innovative biocompatible, tricalcium cement, is used widely in endodontics owing to its advantageous properties. Yet limitations concerning its radiopacity and other handling properties have compelled the development of different material modifications.

**Aim:**

This scoping review aims to map and synthesize the available evidence on the impact of different fillers and additives on the biological, mechanical, and physical properties of Biodentine in endodontics.

**Methodology:**

This review was performed in line with the guidelines of Preferred Reporting Items for Systematic Reviews and Meta‐Analyses Modifications for Scoping Reviews (PRISMA‐ScR) guidelines. A Literature search was conducted comprehensively throughout major databases such as Scopus, PubMed, Web of Science, and Google Scholar. In vitro studies evaluating the modifications of Biodentine were included. Information was charted and integrated descriptively.

**Result:**

Broad range of modifications were noted, such as the addition of nanoparticles, radiopacifiers, bioactive additives. and antimicrobial agents. These modifications were focused on improving the physical and mechanical properties, improve antimicrobial efficacy and enhance radiopacity while preserving biocompatibility. However, significant heterogeneity was noted in modification process, property evaluating methods, and results.

**Conclusion:**

This scoping review indicates that modifications of Biodentine using various additives may enhance its biological, mechanical, and physical properties. Future research should focus on standardizing experimental methodologies to allow meaningful comparison across studies, identifying optimal concentrations of additives to balance material performance, and conducting well‐designed in vivo and clinical studies to validate these findings and support their translation into clinical practice.

## 1. Introduction

The pulp‐dentin complex is a special connective tissue comprised of hard dentin, that forms the major part of the tooth’s structure, and the pulp, which is responsible for nourishing the dentin tissues while providing innervation and defense to the tooth [1]. Carious lesions and trauma are the most prevalent causes of dentin‐pulp complex damage, which, if left untreated, can progress to pulpal disease and pulp necrosis. Endodontic treatment may not be required if early care is obtained [2]. Regenerative therapies such as vital pulp therapy (VPT) varying from minimal to quite intrusive, direct pulp capping, partial pulpotomy, and complete/coronal pulpotomy have acquired popularity in conservative endodontic procedures due to their ability to restore natural structures rather than merely performing as a physical barrier [3].

The pulp and surrounding tissues should be conserved and regenerated by the absolute endodontic material that can ensure the long‐term effectiveness of the treatment and ultimately lead to a healthy and functional dentition [4]. Calcium silicate‐derived cements are among the numerous bioactive materials that were developed to interact positively with living tissues. Biodentine (Septodont, Saint‐Maur‐des‐Fossés, France) is a notable and widely used calcium silicate cement. It is frequently referred to as a “dentin replacement material” [5]. Biodentine is a bioactive, fast‐setting material that can provoke a positive reaction from the host tissue. Additionally, it can release calcium and indicates bio‐interactivity. It induces reparative dentin synthesis; furthermore, it is alkaline with good physical and mechanical properties [6].

**Figure 1:**
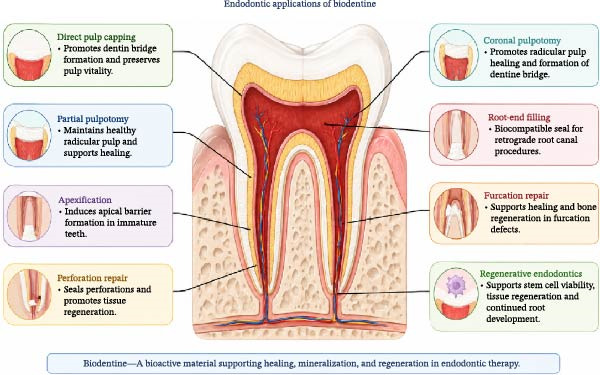
Endodontic applications of Biodentine.

Owing to its dentin‐like mechanical properties, it possesses a wide range of uses in endodontics (Figure 1). It acts as a dentin replacement in deep cavities for composite repair [7]. Biodentine is used as an indirect pulp capping material mainly because it can promote reactive dentin formation. It is often used in vital pulp treatments such as pulpotomy, pulpectomy, and pediatric dentistry [8]. Further, it is recommended for perforation and furcation repair, as well as a retrograde filling material [9]. In addition to its conventional applications, Biodentine has also gained considerable attention in regenerative endodontic procedures due to its bioactive properties, ability to promote mineralization, and favorable interaction with stem cells, thereby supporting tissue regeneration [10].

Despite these advantages, Biodentine exhibits certain limitations, including relatively low radiopacity, handling challenges, and susceptibility to washout [11]. To address these issues, various modifications have been proposed. The incorporation of radiopacifiers such as zirconium‐ or ytterbium‐based compounds has been investigated to improve radiographic visibility [12]. Handling characteristics and flow have been enhanced through the addition of polymers and organic agents [13], while reinforcement strategies using nanoparticles, fibers, and bioactive glass have been explored to improve mechanical strength and reduce washout [14]. Furthermore, the incorporation of nanomaterials such as graphene, zirconia, and bioglass has been shown to enhance physicochemical stability and bioactivity [15]. These targeted modifications aim to optimize Biodentine’s performance for diverse clinical applications.

Nanotechnology is a developing domain of material science with dimensions of no more than 100 nm. It impacted the dental and medical fields by enhancing the mechanical and physical characteristics of materials, enabling the development of new diagnostic modalities and nano‐delivery systems [11]. The application of nanotechnology in endodontics is convincing. It mainly includes adding various nanoparticles like zirconia, glass ceramics, graphene, and bioglass to endodontic material and sealers. The use of nanoparticles improves the adaptation of material in addition to its setting time, chemical bonding, dimensional stability, and osteoconductivity [16].

Despite various studies having researched these modifications individually, a review of the impact of different fillers on the properties of Biodentine is still lacking. Understanding these tendencies is essential for the development of future materials and their clinical applications. Therefore, the present scoping review aims to map and synthesize the available evidence on the impact of different fillers and additives on the biological, mechanical, and physical properties of Biodentine in endodontics.

## 2. Methodology

The scoping review was carried out in compliance with the Arksey and O’Malley approach [17] and aligned to the Preferred Reporting Items for Systematic Reviews and Meta‐Analyses Modification for Scoping Reviews (PRISMA‐ScR) guidelines [18].

### 2.1. Analyzing the Research Question

The fundamental question of research was “How do modifications with different fillers or additives affect the biological, physical, and mechanical properties of Biodentine in endodontics?” The population–concept–context (PCC) model guided the formulation of this question.

### 2.2. Finding Relevant Studies

#### 2.2.1. Search Methodology

A comprehensive search was performed to identify all available literature investigating modified forms of Biodentine incorporated with nanoparticles, radiopacifiers, bioactive glasses, polymers, reinforcing fibers, or other fillers. Studies addressing variations in Biodentine’s features both in vitro and ex vivo were accepted for participation. There were, however, no limitations on published years, and solely studies in the English language have been taken into consideration. Electronic search was performed in PubMed, Scopus, Web of Science, and Google Scholar. For Google Scholar, all retrieved records were screened without applying any restriction based on ranking or relevance order, ensuring comprehensive coverage of potentially eligible studies. Manual screening of references was additionally done by looking at the bibliographies of the studies that were included along with applicable reviews to make certain that no significant research studies were overlooked throughout the database searches.

#### 2.2.2. Search Terms

The search strategy implemented keywords associated with Medical Subject Headings (MeSH) terms to uncover studies addressing biodentine alterations and effects they have on the properties of material in endodontics.

Key terms relevant to the biomaterial were “Bioceramic Materials,” “Calcium Silicate Cement,” “Tricalcium Silicate,” and “Biodentine.” “Nanoparticles,” “Additives,” “Reinforcement,” “Fillers,” “Graphene,” “Bioactive Glass,” “Zirconia,” and “Radiopacifiers” were the terms related to modification.

Keywords including “Biological Properties,” “Physical Properties,” “Mechanical Properties,” “Bioactivity,” “Setting Time,” “Microhardness,” “Cell Viability,” and “Compressive Strength” were used to locate research pertinent to assessment of properties. The terms used in the endodontic context were “Endodontics,” “Pulp Therapy,” “Vital Pulp Therapy,” “Root Repair Materials,” and “Endodontic Materials.”

Boolean operators such as AND and OR were implemented for combining the key terms and revised to the indexing criteria of individual database. The keywords of the search were developed in association with an expert like a medical librarian in scoping and systematic review analyses to improve accuracy, sensitivity, and specificity. Table 1 summarizes the extensive search strategies and their corresponding outcomes.

**Table 1:** Methods of investigative strategies and data resources.

Database	Research strategies	Results
PubMed	(“Biodentine” OR “calcium silicate cement” OR “tricalcium silicate” OR “bioceramic material”) AND (“modification” OR “nanoparticle” OR “filler” OR “additive” OR “reinforcement” OR “bioactive glass” OR “zirconia” OR “graphene” OR “radiopacifier”) AND (“mechanical property” OR “physical property” OR “biological property” OR “microhardness” OR “setting time” OR “compressive strength” OR “cell viability”)	245
Google Scholar	“Biodentine modification” OR “Biodentine nanoparticle” OR “modified Biodentine” OR “Biodentine reinforced” OR “nanoparticle added to Biodentine”	420
Scopus	TITLE‐ABS‐KEY (“Biodentine” OR “calcium silicate cement” OR “tricalcium silicate” OR “bioceramic material”) AND TITLE‐ABS‐KEY (“nanoparticle” OR “filler” OR “additive” OR “reinforcement” OR “bioactive glass” OR “zirconia” OR “graphene” OR “radiopacifier”) AND TITLE‐ABS‐KEY (“mechanical property” OR “physical property” OR “biological property”)	165
Web of Science	TS = (“Biodentine” OR “calcium silicate cement” OR “bioceramic material”) AND TS = (“nanoparticle” OR “filler” OR “additive” OR “reinforcement” OR “bioactive glass” OR “zirconia” OR “graphene” OR “radiopacifier”) AND TS = (“mechanical property” OR “physical property” OR “biological property”)	120
Total	—	1000

### 2.3. Inclusion and Elimination Parameters

Research studies were considered provided they (1) investigated Biodentine incorporated with nanoparticles, radiopacifiers, fillers, polymer fibers, or any other additive; (2) assessed biological, physical, or mechanical properties related to endodontic use; and (3) were original studies (in vitro, ex vivo, or laboratory‐based). Reviews, letters, case reports, abstracts devoid of full text, editorials, research work published apart from the English language, animal studies, and the others lacking Biodentine modifications.

### 2.4. Identifying Relevant Studies

The retrieved information was entered into reference‐managing software, followed by the elimination of duplicates. Two individual reviewers thoroughly screened both titles and abstracts for acceptability using predefined requirements. Disagreements were resolved by discussion or by obtaining some advice from a third reviewer.

#### 2.4.1. Screening Process

**Figure 2:**
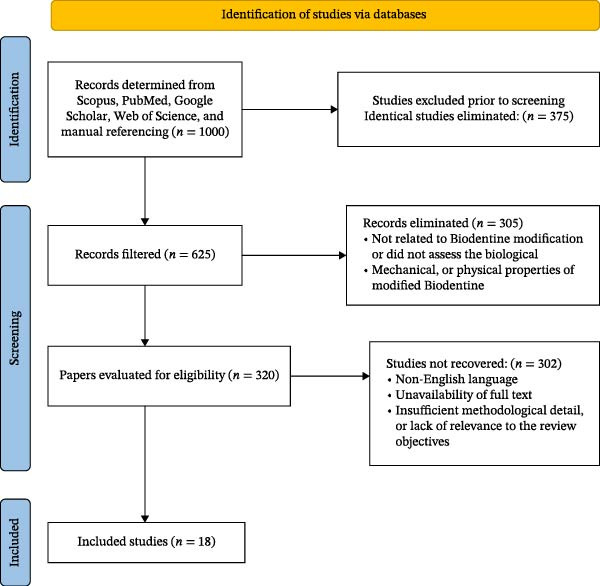
PRISMA flowchart shows the process of screening of obtained studies from various databases.

Two phases formed the verification process, which included (1) review of the heading and abstract and (2) full‐text assessment of possible appropriate research studies. Full‐text studies were evaluated against the inclusion criteria, and reasons for exclusion were recorded. The entire study selection process is shown in a PRISMA flow diagram (Figure 2).

### 2.5. Charting the Information

Data retrieval was carried out using a standardized data‐charting framework designed by the authors. Extracted details were author details, year of publication, type of filler used, study design, experimental methods, outcome measures, and key findings.

### 2.6. Data Synthesis

Collected data was classified descriptively and categorized based on the type of modification introduced. The results obtained were classified into three main categories: mechanical characteristics (e.g., microhardness and compressive strength), physical features (e.g., solubility, setting period, and radiopacity), and biological effects (e.g., bioactivity and cell longevity). Due to heterogeneity among research study designs and conclusions, meta‐analysis was not carried out.

### 2.7. PCC Framework

The PCC framework was adopted to determine eligibility and improve transparency in the review procedure.

• Population: Studies with Biodentine or Biodentine‐based formulations.
• Concept: Modifications using nanoparticles, radiopacifiers, fillers, polymers, fibers or additional additives are indicated to improve the properties of materials.
• Context: Endodontic applications and laboratory investigations are applicable to clinical use.

### 2.8. Consultation

An expert consultation step was included to enhance the comprehensiveness and validity of the review. Two endodontic specialists with academic and research experience in calcium silicate‐based materials were consulted. Their role involved reviewing the search strategy, verifying the relevance of included studies, identifying any potentially missed literature, and providing insights into the interpretation of findings. Feedback from the experts contributed to refining the scope of the review, ensuring the completeness of the included evidence, and improving the clinical relevance of the final synthesis.

## 3. Result

### 3.1. Selection of Research Studies

A total of 1000 records were found from Scopus, PubMed, Web of Science, Google Scholar, and manual reference assessment of related dental journals. After eliminating 375 duplicates, 625 records were reviewed by title and abstract. Of these, 305 studies were eliminated since they were irrelevant to Biodentine modification or failure to evaluate the biological, physical, or mechanical properties of altered Biodentine. Out of the remaining 320 articles, 302 were excluded after full‐text analysis due to considerations like non‐English language, lack of full text, inadequate methodological detail, or being irrelevant to the review objectives. Eventually, 18 studies published from 2000 to 2025 fulfilled the requirements of inclusion and were accordingly incorporated into this scoping review.

Ultimately, 18 studies, published between 2000 and 2025, matched the selection criteria and were thus incorporated into the scoping review. The entire procedure for study selection is demonstrated in the PRISMA‐ScR flowchart (Figure 2).

### 3.2. Characteristics of Studies Included

**Figure 3:**
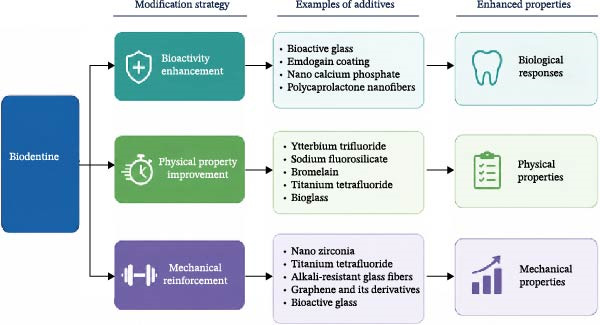
Modifications of Biodentine and their effect on its properties.

This review included 18 in vitro studies published between 2000 and 2025. All studies examined alterations made to Biodentine through the addition of different nanoparticles, fillers, polymers, radiopacifiers, or additives to improve its biological, physical, or mechanical properties (Figure 3). Early studies focused on enhancing biomineralization, such as incorporation of nano calcium phosphate, which was associated with increased dentin bridge formation [19]. Subsequent studies addressed reinforcement techniques, such as alkali‐resistant glass fibers, that improved compressive and tensile strength [20] and nanoparticles of bioactive glass, which increased interfacial bonding and apatite formation [21, 22].

Ytterbium trifluoride was employed in later studies to improve physicochemical properties including radiopacity [12], and the addition of titanium tetrafluoride to enhance antibacterial activity, fracture resistance, and microhardness [23]. Incorporation of Emdogain was one of the biological improvement strategies that stimulated odontogenic gene expression and stem cell mineralization [24]. Furthermore, scaffold‐based methods were developed, with Biodentine‐coated polycaprolactone nanofibers enhancing biomineralization [25].

Recent studies focused on advanced nanomaterials, such as fluorinated graphene [26], nano‐zirconia [27], graphene oxide [28], and different bioactive glass nanocomposites [29, 30]. These studies showed changes in bond strength, mineralization ability, ion release, and mechanical hardness. Additional modifications intended at enhancing handling properties, such as adding bromelain, that improved the flowability of Biodentine without reducing strength [31], and also PMMA‐nanoclay, which greatly increased early‐stage adhesion to resin composites [32].

### 3.3. Summary of Findings Across Major Domains

#### 3.3.1. Mechanical Properties

Various additives are associated with increased mechanical performance, specifically microhardness, tensile strength, compressive strength, and bond strength. Microhardness and water sorption were improved by 10%–20% nano‐zirconia, with 20% presenting the best entire mechanical profile [27]. Further, hardness, fracture resistance, and tensile strength were enhanced by titanium tetrafluoride (1–2 wt%) [23], while alkali‐resistant glass fibers (5%) reinforced Biodentine and elevated vertical fracture resistance when added as an intraorifice barrier [20]. Graphene oxide (1%) decreased setting time and improved push–out bond strength [28], while fluorinated graphene greatly enhanced compressive strength [26]. The addition of bioactive glass further improved microhardness [29]. The majority of studies found that modifications had no impact on the fundamental strength of Biodentine, while several provided significant improvements.

#### 3.3.2. Physical Properties

Physical features like solubility, setting time, ion release, and radiopacity were increased by particular fillers. Ytterbium trifluoride (2.5%–7.5%) increased radiopacity, particularly 2.5% offering a favorable balance of setting time and physical durability [12]. Fluoride‐releasing modifications, including sodium fluorosilicate and hydrofluoric acid elevated the release of fluoride while maintaining the compressive strength [33]. Setting time and solubility were decreased by nano‐bioactive glass [29], while bromelain enhanced flowability without compromising mechanical stability [31]. Furthermore, titanium tetrafluoride and bioglass formulations enhanced radiopacity [23, 34]. Certain modifications, like higher concentrations of TiF_4_, elongated the setting time, indicating the significance of an ideal dosage.

#### 3.3.3. Biological Responses

Biological effects of altered Biodentine were assessed through cell viability, ALP functioning, mineralization evaluation, and apatite formation. Fluorinated graphene stimulated the development of hydroxyapatite while retaining biocompatibility by increasing the pH and releasing calcium ions [26]. Nanoparticles of bioactive glass constantly accelerated apatite formation and improved dentin‐cement surface properties [21, 22]. Odontogenic markers (DSPP, DMP1, and BSP) were upregulated, and mineralized nodule formation was increased by emdogain‐coated Biodentine [24]. Scaffold‐related modifications including Biodentine‐coated PCL nanofibers increased biomineralization and cell adhesion, particularly at 0.01% coating concentrations [25]. In pulp‐capping cases, dentin bridge thickness was increased by nanocalcium phosphate, suggesting early signs of increased reparative dentinogenesis over time [19]. All of these outcomes suggest that specific nanomaterials and bioactive coatings may optimize the biological properties of Biodentine (Table 2).

**Table 2:** Characteristics of Included Studies.

Author (year)	Study design	Filler/additive	Experimental methods	Outcome measures	Key findings
Aidaros et al. (2000) [19]	In vitro	Nanocalcium phosphate (NCP)	Pulp exposure; histology; SEM‐EDX	Dentin bridge thickness; Ca/P content	Thicker dentin bridge; enhanced dentinogenesis
Nagas et al. (2016) [20]	In vitro	AR glass fibers (5%, 10%)	DTS; compressive strength; fracture resistance	Mechanical properties	5% fibers improved strength; highest fracture resistance with BD + 5% fibers
Corral Nuñez et al. (2017) [21]	In vitro	Bioactive glass (BG) nanoparticles (1%, 2%)	SBF immersion; XRD; FTIR; SEM‐EDX	Apatite formation; interface	Faster apatite formation; improved bonding
Simila et al. (2018) [22]	In vitro	High‐F, High‐Sr F + Sr BG	FTIR; XRD; MAS‐NMR; fluoride release	Apatite type; fluoride release	Enhanced bioactivity; fluorapatite formation
Elsaka et al. (2019) [23]	In vitro	Titanium tetrafluoride (1–3 wt%)	Setting time; DTS; hardness; antibacterial; radiopacity	Mechanical; physical; antibacterial	1%–2% TiF_4_ improved strength and antibacterial effect; 3% ↑ solubility
Karpukhina and Bushby (2021) [34]	In vitro	Fluoride and strontium bioglass	FTIR; XRD; ISO tests; fluoride release	Bioactivity; radiopacity; setting	Improved bioactivity and strength; some delayed setting
Bahaa et al. (2022) [12]	In vitro	YbF3 (2.5%–7.5%)	Radiopacity; strength; setting; pH; ESEM‐EDX	Radiopacity; strength; bioactivity	Radiopacity ↑; 2.5% best balance; alkaline pH maintained
Kamal et al. (2022) [35]	In vitro	BG	Furcation dye penetration	Sealing ability	BG improved BD sealing; MTA best overall
Karkehabadi et al. (2022) [24]	In vitro	Emdogain coating	MTT; qRT‐PCR (DSPP, DMP1, BSP); ALP; mineralization	Viability; gene expression; ALP	BD + Emdogain ↑ odontogenic markers and mineralization
Sekhar et al. (2023) [33]	In vitro	Na‐fluorosilicate; HF	SPADNS fluoride release; compressive strength	Fluoride release; strength	Fluoride release ↑; strength unchanged
Batul et al. (2023) [27]	In vitro	Nano‐zirconia (10%–30%)	Hardness; sorption; solubility; FTIR	Microhardness; solubility	20% ZrO_2_ best mechanical profile; 30% ↑ solubility
Guneser et al. (2023) [29]	In vitro	Nano‐BG (1%, 2%)	Strength; hardness; setting; solubility	Mechanical and physical	↓ Setting time; ↓ solubility; ↑hardness
Agrawal et al. (2024) [31]	In vitro	Bromelain	ISO tests; strength; solubility; radiopacity; flow	Strength; radiopacity; flow	Strength same; ↑ flow; radiopacity similar
Jagtap et al. (2025) [28]	In vitro	Graphene oxide (1%)	Setting time; push‐out strength	Setting; bond strength	GO ↓ setting time and ↑bond strength
Elmergawy et al. (2025) [26]	In vitro	Fluorinated graphene	XRD; FTIR; SEM; ion release; MTT; ALP; strength	Ion release; pH; viability; strength	↑ Ca^2+^ release; ↑ pH; ↑ strength; biocompatible
Kokate et al. (2025) [30]	In vitro	Nano‐BG (1%–5%)	MTT; ARS; ALP and OSN expression	Viability; mineralization; gene expression	↑ Mineral deposition; gene expression lower vs MTA
Sarvarian et al. (2025) [25]	In vitro	BD‐coated PCL (0.01%–0.05%)	SEM; MTT; ALP; Ca deposition	Viability; mineralization	0.01% best for viability and biomineralization
Elmergawy et al. (2025) [32]	In vitro	PMMA–nanoclay (10%)	XRD; FTIR; SEM‐EDX; microshear strength	Bond strength	↑ Early bond strength; no change at 2 h/2 weeks

## 4. Discussion

This scoping review synthesizes evidence from 18 in vitro studies investigating how various additives influence the biological, mechanical, and physical properties of Biodentine. Rather than merely reporting outcomes, the collective evidence highlights a clear shift toward material engineering approaches aimed at optimizing Biodentine’s clinical performance. The modifications explored in these studies target specific limitations of conventional Biodentine such as suboptimal radiopacity, handling challenges, and susceptibility to solubility, while simultaneously enhancing its bioactivity and structural integrity. Importantly, the findings suggest that improvements are not isolated to single properties; instead, many additives exert multifactorial effects, influencing the biological response, physicochemical behavior, and mechanical stability simultaneously.

A key pattern emerging across studies is the role of nanostructured bioactive materials in enhancing the regenerative potential of Biodentine. Bioactive glass formulations, particularly fluoride‐ and strontium‐containing variants, consistently demonstrated accelerated apatite formation and improved dentin–cement interface characteristics [21, 22]. These effects can be attributed to enhanced ion exchange and surface reactivity, which promote the early nucleation of calcium phosphate phases. Similarly, calcium‐rich additives such as nanocalcium phosphate [19] and advanced nanomaterials like fluorinated graphene [26] appear to modulate ionic release and local alkalinity, thereby creating a favorable microenvironment for mineralization. Collectively, these findings support the concept that bioactivity in calcium silicate cements can be strategically amplified through nanoscale modifications, which may have direct implications for improving outcomes in VPT and regenerative endodontics.

In parallel, several studies demonstrated that mechanical reinforcement is achievable through the targeted incorporation of reinforcing agents. Materials such as alkali‐resistant glass fibers [20] and nano‐zirconia [27] function as structural stabilizers within the cement matrix, improving resistance to fracture and deformation under functional loads. Graphene‐based additives, including graphene oxide and fluorinated graphene [26, 28], introduce an additional dimension of reinforcement by enhancing interfacial bonding and distributing stress more effectively due to their high‐surface area and mechanical strength. However, an important observation across studies is that mechanical enhancement is highly dependent on concentration, with optimal ranges providing reinforcement, whereas excessive incorporation may disrupt the matrix integrity or adversely affect other properties. This highlights the need for careful formulation design rather than the indiscriminate addition of reinforcing agents.

From a clinical perspective, improvements in physical properties are particularly relevant as they directly influence handling, placement, and radiographic evaluation. Radiopacity, a known limitation of Biodentine, was effectively enhanced through the incorporation of radiopacifiers such as ytterbium trifluoride and titanium tetrafluoride [12, 23]. Similarly, modifications involving nano‐bioactive glass and graphene derivatives demonstrated reduced setting times, which could facilitate more efficient clinical workflows [28, 29]. Adjustments in handling properties, such as increased flow with bromelain [31] and improved early bonding with PMMA‐nanoclay systems [32], further illustrate how material modifications can address practical clinical challenges. These findings suggest that customizable formulations of Biodentine may allow clinicians to tailor material properties according to specific procedural requirements. However, given that the current evidence is derived from in vitro studies, caution must be exercised when extrapolating these findings to clinical scenarios, and further in vivo and clinical investigations are necessary to confirm their applicability.

The observed modifications may have important implications for clinical practice. Enhanced bioactivity and mineralization potential suggest possible benefits in VPT and regenerative endodontic procedures, where improved tissue response and dentin formation are critical [19, 21, 25]. Improvements in mechanical properties may support the use of modified Biodentine in stress‐bearing applications such as perforation repair and intraorifice barriers [20, 27, 28]. Additionally, increased radiopacity facilitates more reliable radiographic assessment [12, 23], while reduced setting time and improved handling characteristics may enhance procedural efficiency [29, 31]. Modifications that improve interfacial bonding may also contribute to better integration with restorative materials [32]. However, as these findings are based on in vitro studies, their clinical applicability should be interpreted with caution, and further in vivo and clinical investigations are required.

A comparative synthesis of the included studies indicates that specific categories of modifications demonstrate relatively consistent effects. Bioactive glass‐based additives were commonly associated with enhanced bioactivity and apatite formation [21, 22, 25], whereas graphene‐based materials showed consistent improvements in mechanical properties and ion release [26, 28]. Reinforcing agents such as nano‐zirconia and glass fibers primarily contributed to mechanical stability [20, 27], while radiopacifiers improved radiographic visibility without markedly affecting biological performance [12, 23]. However, these effects were influenced by additive concentration and formulation, with optimal ranges yielding favorable outcomes and higher concentrations occasionally resulting in adverse effects such as increased solubility or delayed setting [23, 27, 30]. These observations highlight the need to balance multiple material properties and emphasize the importance of standardized methodologies for meaningful comparisons across studies.

Another important insight from this review is the interdependence of material properties. Modifications designed to enhance one property often influence others, either positively or negatively. For example, while certain additives improved mechanical strength or bioactivity, higher concentrations were sometimes associated with increased solubility, delayed setting, or reduced cell viability [23, 27, 30]. This reinforces the concept that Biodentine modification should not be viewed as a single‐parameter optimization problem but rather as a multidimensional balance between biological compatibility, mechanical stability, and physicochemical performance.

A notable limitation across the included studies was the considerable heterogeneity in the study design and methodology. Variations were observed in the type and concentration of additives used (e.g., nanoparticles, bioactive glass, polymers, and radiopacifiers), as well as in preparation protocols, mixing techniques, and storage conditions. Additionally, differences in experimental models, testing standards, and outcome measures such as methods for assessing microhardness, compressive strength, ion release, and bioactivity further limited direct comparison between studies. This methodological variability reduces the ability to draw definitive conclusions or perform quantitative synthesis, emphasizing the need for standardized testing protocols in future research.

### 4.1. Limitations

Despite the positive findings, a number of gaps and shortcomings were identified. Since every listed study in this review was in vitro, it restricts the translation of outcomes to clinical applications. It is challenging to accurately replicate features such as moisture contamination, inflammatory response, blood flow, and prolonged mechanical stress in a laboratory environment. Further, there were significant methodological differences throughout the studies like differences in additive dosage, incubation environment, mixing techniques, sample preparation, and outcome evaluation techniques, complicating direct comparison and analysis. Standardized methods would enhance future research and enable more reliable cross‐study comparisons. Also, few research studies investigated durability over the long term, aging behavior, or relationship to restorative materials—all of which are essential considerations for the clinical longevity of Biodentine. In addition, the relatively small number of included studies limits the generalizability of the findings and highlights the need for further well‐designed investigations in this field. Furthermore, as this review was conducted as a scoping review, no formal quality assessment or risk‐of‐bias evaluation of the included studies was performed, which should be considered when interpreting the findings.

## 5. Conclusion

This scoping review indicates that modifications of Biodentine using various additives may enhance its biological, mechanical, and physical properties. Despite the results being dose‐dependent, most of the modifications improved performance without impairing biocompatibility. Even with promising laboratory outcomes, clinical applicability remains uncertain owing to limited standardization and limited in vivo research. Future research should focus on standardizing experimental methodologies to allow meaningful comparison across studies, identifying optimal concentrations of additives to balance material performance, and conducting well‐designed in vivo and clinical studies to validate these findings and support their translation into clinical practice.

## Author Contributions

Rumesa Batul, Abdul Habeeb Adil, and Niher Tabassum Snigdha contributed to investigation, writing – original draft, review and editing, and data collection. Ankita Mathur and Sushma Bommanavar contributed to writing, review and editing, and figure editing. Mohmed Isaqali Karobari contributed to conceptualization, investigation, writing – original draft, review and editing, supervision, and project administration. Mohmed Isaqali Karobari (corresponding author) had full access to all of the data in this study and takes complete responsibility for the integrity of the data and the accuracy of the data analysis.

## Funding

This study did not receive any specific funding.

## Disclosure

All authors contributed critically to the manuscript, approved the final version, and have read and agreed to the published version of the manuscript.

## Ethics Statement

This study is a scoping review of previously published literature and did not involve human participants, patient data, or any intervention. Therefore, ethical approval was not required.

## Conflicts of Interest

The authors declare no conflicts of interest.

## Data Availability

The authors confirm that the data supporting the findings of this study are available within the article and/or its Supporting Information.
